# Supplementary material for: Integrated analysis of the aqueous humor microbiome and lens capsule transcriptome in high myopia cataract: a pilot study
Source: Front Med (Lausanne). 2026 Jun 16;13:1845205. doi: 10.3389/fmed.2026.1845205 (PMC13314463; doi:10.3389/fmed.2026.1845205)
Supplement: Supplementary file 3 [file Data_Sheet_1.zip › 4.Meta_Statistic/C372089/LEfSe/biomarkers_raw_images/1_o__Flavobacteriales.pdf]

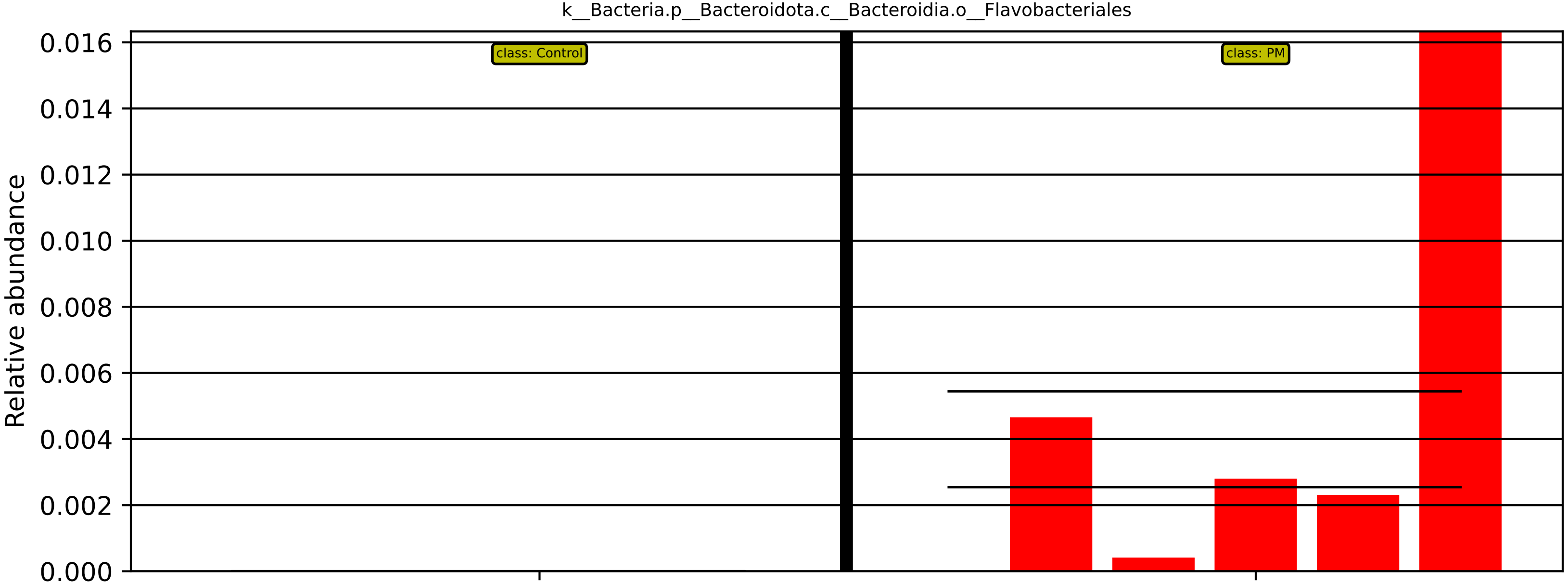
k__Bacteria.p__Bacteroidota.c__Bacteroidia.o__Flavobacteriales
class: Control
class: PM
Relative abundance
0.016
0.014
0.012
0.010
0.008
0.006
0.004
0.002
0.000
